# Rapid differentiation of sexual signals in invasive toads: call variation among populations

**DOI:** 10.1038/srep28158

**Published:** 2016-06-22

**Authors:** Kiyomi Yasumiba, Richard L. Duffy, Scott A. Parsons, Ross A. Alford, Lin Schwarzkopf

**Affiliations:** 1 College of Marine and Environmental Sciences, James Cook University, Townsville, QLD, Australia

## Abstract

Advertisement calls tend to differ among populations, based on morphological and environmental factors, or simply geographic distance, in many taxa. Invasive cane toads (*Rhinella marina*) were introduced to Australia in 1935 and their distribution has expanded at increasing rates over time. Rapid evolution occurred in morphological and behavioural characters that accelerate dispersal, but the effects of rapid expansion on sexual signals have not been examined. We collected advertisement calls from four populations of different ages since invasion, and analysed the geographic differentiation of seven call parameters. Our comparisons indicate that the calls of *R. marina* differ among Australian populations. The signal variation was not simply clinal with respect to population age, climate, or morphological differentiation. We suggest that selection on signalling among populations has been idiosyncratic and may reflect local female preferences or adaptation to environmental factors that are not clinal such as energy availability.

Anuran advertisement calls are classic examples of sexually selected characters^1^,^2^. Advertisement call variation is often correlated with aspects of male quality, such as body size, providing “honest signals” of quality and a basis for female mate choice^2^,^3^. For example, larger males tend to produce calls with lower dominant frequencies, which are often preferred by females^4^,^5^,^6^. In addition, because they are ectotherms, anuran call characteristics are influenced by environmental factors^2^,^7^,^8^,^9^, e.g., pulse rate, which is affected by muscle contraction rates, is strongly constrained by temperature^1^,^10^. Divergence among populations within species is often geographically clinal; these clines are almost always attributed to morphological, environmental or genetic factors including body size, geographic distance, climate variation, and genetic drift^11^,^12^. To date, most studies on signalling variation among populations of animals with acoustic sexual signals have examined populations within species’ natural ranges^13^,^14^,^15^.

Australian populations of the cane toad, *Rhinella marina*, present an opportunity to examine signal variation among recently established populations with relatively well-documented histories of spread. This highly invasive species spread rapidly from eastern to far northern and north-western Australia since it was introduced along the eastern coastline in 1935^16^,^17^. During the course of the cane toad invasion, over about 80 toad generations^16^, morphology and behaviour have evolved rapidly to facilitate dispersal^18^,^19^,^20^. These changes have included increases in relative leg length^18^ and modifications to dispersal behaviour; individuals move further and more often^19^,^20^.

Here, we investigated whether a sexual signal, the mating call of cane toads has differentiated among populations of different ages in Australia. Toad populations in Townsville (north-eastern Queensland; NEQ) were established by 1940, and in Brisbane (south Queensland; SQ), were established by 1955^21^, whereas populations in Longreach (central Queensland; CQ) and Kununurra (western Australia; WA) arrived more recently ca. 2006^22^ and 2010^23^,^24^ (Fig. 1). There is relatively little genetic differentiation among populations of cane toads^25^,^26^, and therefore it is interesting to explore whether acoustic signals differ among toad populations. We tested the null hypothesis that the characteristics of calls did not differ among these populations. In addition, because these populations are widespread geographically, and exposed to quite different environments, we determined whether variation among populations was correlated with differences in morphology, or with clines in environmental characteristics. Finally, because there is variation in the time since establishment of these populations, we examined variation in call characteristics in relation to time since invasion.

## Results

Our initial multivariate analysis indicated that there is significant geographic variation in call properties among populations (MANOVA; Wilk’s lambda = 0.472; approximate *F*_3,96_ = 3.686; *P* < 0.001; Fig. 2). Subsequent pairwise multivariate comparisons revealed that calls of NEQ individuals differed significantly from all other populations (vs SQ; Wilk’s lambda = 0.528; approximate *F*_1,42_ = 4.602; *P* < 0.001, vs CQ; Wilk’s lambda = 0.670; approximate *F*_1,58_ = 3.665; *P* = 0.003, vs WA; Wilk’s lambda = 0.502; approximate *F*_1,58_ = 7.373; *P* < 0.001). The other older population, SQ, did not differ significantly from newer populations, CQ and WA. However, the two newer populations differed significantly from each other (Wilk’s lambda = 0.628; approximate *F*_1,54_ = 4.056; *P* = 0.002).

We followed the multivariate comparisons with univariate comparisons of each call parameter among populations. Most parameters differed significantly among populations (Table 1). The NEQ population had higher dominant frequency, longer call duration with longer pulse length and longer inter-pulse interval than the others (Fig. 2; Table 1). The three remaining populations were all relatively similar in these parameters, but with relatively lower dominant frequencies and shorter inter-pulse intervals than the NEQ population (Fig. 2; Table 1). The SQ population was near the mean for the remaining parameters, pulse length and call duration, however the CQ and WA populations were significantly differentiated along this axis; CQ toads had relatively greater pulse lengths and shorter call durations, while WA toads diverged in the opposite direction, with shorter pulse lengths and longer call durations (Fig. 2; Table 1).

Calls vary among individuals as well as among populations, so we examined the among- versus within-population variation in individual call parameters. Dominant frequency and pulse rate varied little among individuals within populations, as a static property, typically less than about 5%^27^ (Table 2). Inter-call interval length showed the highest variability among individuals within populations (Table 2). The variation in dominant frequency, call duration, inter-call interval, and inter-pulse interval, were higher among than within populations (i.e., values of CVa/CVp, were higher) (Table 2), whereas the variation in proportion of time spent calling and pulse rate were about equal within and among populations (as shown in Table 1). Pulse length showed the largest ratio of overall to within-population variation (Table 2).

Differences in time since colonisation among sites, or climate gradients among sites could have caused clinal variation in call parameters. Given the locations of the sites, our results were not consistent with the existence of a gradient related to time since colonisation (*r*^2^ = −0.248, *F*_1,4_ = 0.006, *P* = 0.944). In addition, none of the distances between sites in terms of eight different climatic variables (mean annual, maximal and minimal temperatures, mean temperature warmest and coldest quarters, mean annual rainfall, temperature seasonality and rainfall seasonality) were significantly correlated with the distances in call parameters between locations; the most highly correlated climate factor was mean annual temperature, explaining 25% of the variation in call parameters between populations, which was not significant (*r*^2^ = −0.250, *F*_1,4_ < 0.001, *P* = 0.992).

## Discussion

The acoustic signals of invasive cane toads differed significantly among populations colonized at different times. This geographic variation indicates that there has been rapid differentiation of call properties since the initial introduction of toads to Australia, and its pattern indicates that differentiation has not been not simply clinal, driven by environmental factors, or related to the differentiation in other characters that has resulted from spatial sorting.

Most parameters, except for pulse rate and proportion of time spent calling, differed significantly among populations after we removed size and temperature effects (Table 1). Our examination of relative levels of variability in parameters indicated that dominant frequency and pulse rate varied less within populations. Gerhardt^27^ suggested that call properties with low levels of within-population variation may be those that are strongly maintained by female choice. Our comparisons of the ratios between among- and within-population coefficients of variation, however, indicated that dominant frequency had relatively high levels of variation among populations. These results suggest that dominant frequency is strongly selected within populations, in directions that differ among populations. Pulse rate, on the other hand, tended not to differ among populations and also varied little within individuals. This call trait often differs among species, driven by evolutionary differentiation for species recognition^1^. Given the absence of closely related frog species within toad distribution in Australia, or even species that sound like them^28^, there may be little selective pressure for species-level differentiation. The degree of variability of this parameter (a range of 10 to 20 pulses/sec) may be limited by morphology. Morphological constraints and/or very strong and consistent sexual selection could account for the relatively low levels of variation of this trait at every level. Proportion of time spent calling did not quite differ significantly among populations, and was relatively less variable among populations than within populations than were call duration and inter-call interval. Thus, proportion of time spent calling has some degree of flexibility within populations, but this flexibility is similar across populations. The highest ratio of among-population to within-population CVs occurred in pulse length, suggesting that variation in this call trait may be strongly reduced by selection within populations, while diverging among populations.

**Figure 1:**
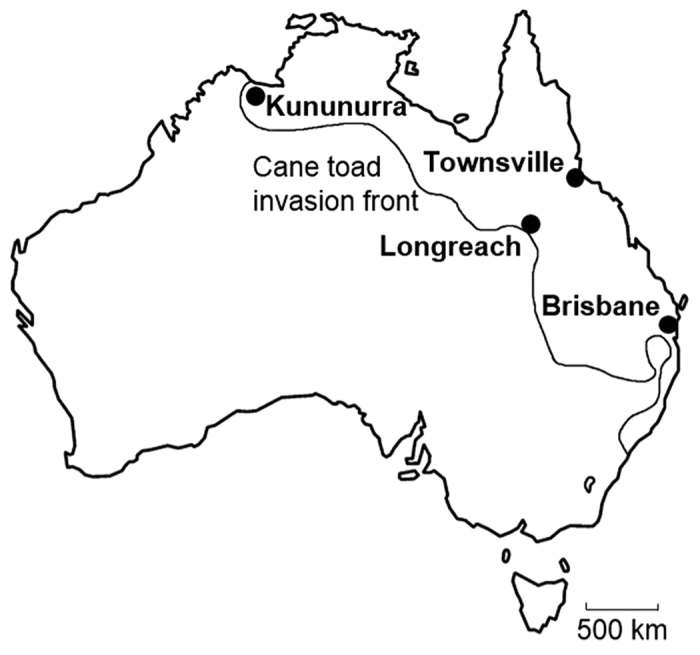
Australia, showing of study locations and cane toad distribution area. Populations sampled were: Townsville (NEQ), Brisbane (SQ), Longreach (CQ), and Kununurra (WA). The line indicates the approximate location of the cane toad invasion front, based on Kearney *et al.*^23^. This figure was created using Microsoft Paint (ver. 6.3, 2013).

**Figure 2:**
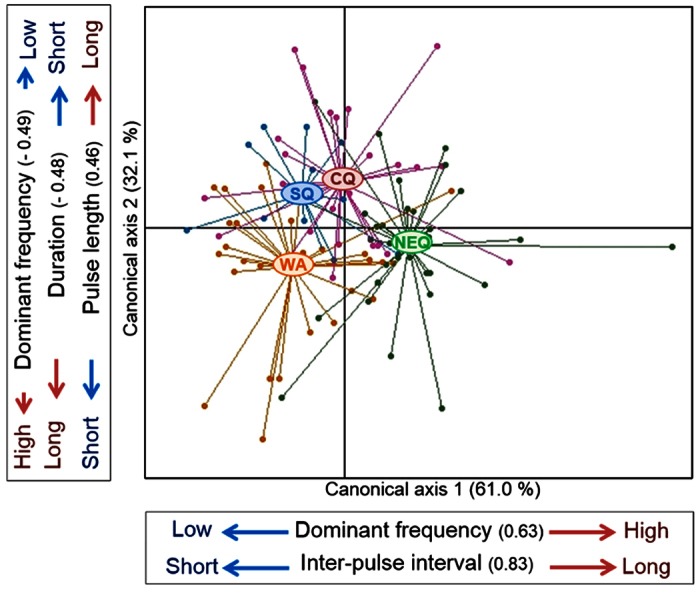
Results of a canonical discriminant function analysis of seven call parameters for each individual in each population. The first (highly significant) axis accounted for 61.0% and the second axis accounted for 32.1% of the total variation among sites. Call parameters with correlations greater than 0.45 of standardised coefficient for each canonical axis are indicated in the text boxes along each axis. The first axis was correlated strongly with inter-pulse interval length and dominant frequency and the second axis was most strongly correlated with dominant frequency, call duration and pulse length.

Another invasive anuran, the Coqui frog (*Eleutherodactylus coqui*) showed differences in call properties among populations at different elevations in its introduced range in Hawaii after about 34.5 generations, but these differences were caused by differences in body size and temperature^29^. Differences among populations in terms of dominant frequency and pulse rate, for instance, can generally be explained by morphological differences (body size or mass) and environmental temperatures, respectively^1^. Our data, however, do not support the hypothesis that cane toad sexual signals varied along simple clinal gradients of temperature or rainfall, as reported in many studies of other anurans^13^,^14^,^15^. The call differentiation we observed among old and new populations with similar colonisation times (Fig. 1) does not suggest spatial or temporal clines caused by toad arrival times (or geographic distance). Similarly, pure drift based on geographic or temporal distance does not account for the similarity between SQ and the newer populations. Thus, the call variation we observed does not seem to be driven by broad clinal patterns of environmental characteristics or by genetic drift related to time since colonisation.

Divergence in female choice among sites could have driven the differences in male signals we observed among populations^30^. Alternatively, this divergence may be a response to selection caused by non-clinal habitat heterogeneity in environmental conditions, including factors affecting signal propagation in different habitats. In addition, metabolic or energetic resources allocated to signalling may influence the character of the signal^13^, and the allocation of resources to signalling may vary among populations as a function of resource availability, or population density, or both^31^,^32^.

In summary, our study revealed significant divergence in calls among populations of toads within their invasive range. Our study suggests that, like morphology and dispersal behaviour, sexual signals have also experienced rapid evolutionary changes. Those changes, however, do not appear to be the product of spatial sorting but may instead reflect adaptation to localised sets of behavioural, ecological or environmental conditions. Local modification of acoustic signals in invasive animals may accelerate population growth and facilitate invasion by improving mating success.

## Methods

### Ethics statement

All procedures in this study were approved by the Animal Ethics Committee at James Cook University (permit no. A1838). All procedures undertaken were in accordance with approved guidelines.

### Recording of toad advertisement calls

Advertisement calls of 32 male cane toads in the Townsville region were recorded in March and April 2014 (NEQ; Fig. 1). We sampled calls in the Brisbane region of south-eastern Queensland (SQ), in central western Queensland near Longreach (CQ), and near Kununurra in Western Australia (WA; Fig. 1). Toad advertisement calls from 12 males in SQ, 28 males in CQ and 28 males in WA were collected in February and March 2014.

Toad calls were recorded using a Marantz PMD 661 compact digital audio recorder (D&M Professional, Itasca, USA), equipped with a NTG3 shotgun microphone (RØDE, Australia). We recorded consecutive advertisement calls from each male in .wav sound format with 96 kHz sample rate and 24 bit-resolution with manual level adjustment. Immediately following each recording, we captured the focal animal and measured its body temperature to an accuracy of 0.1 °C using a digital non-contact infrared thermometer (QM-7221, DIGITECH, Australia), then placed it in a plastic bag for transport to the laboratory. After we finished all recordings at a site, we measured microhabitat conditions, including water and air temperatures (°C) and relative humidity (%, using a whirling hygrometer P2520, G H Zeal Ltd, UK). In the lab, collected toads were euthanized and frozen. We quantified body condition of each toad by measuring body size as snout-urostyle-length (SUL, mm) using plastic callipers (Pittsburgh, Taiwan), and as body mass (g) using an electronic balance (PM400, Mettler, Switzerland).

### Acoustic analysis

**Figure 3:**
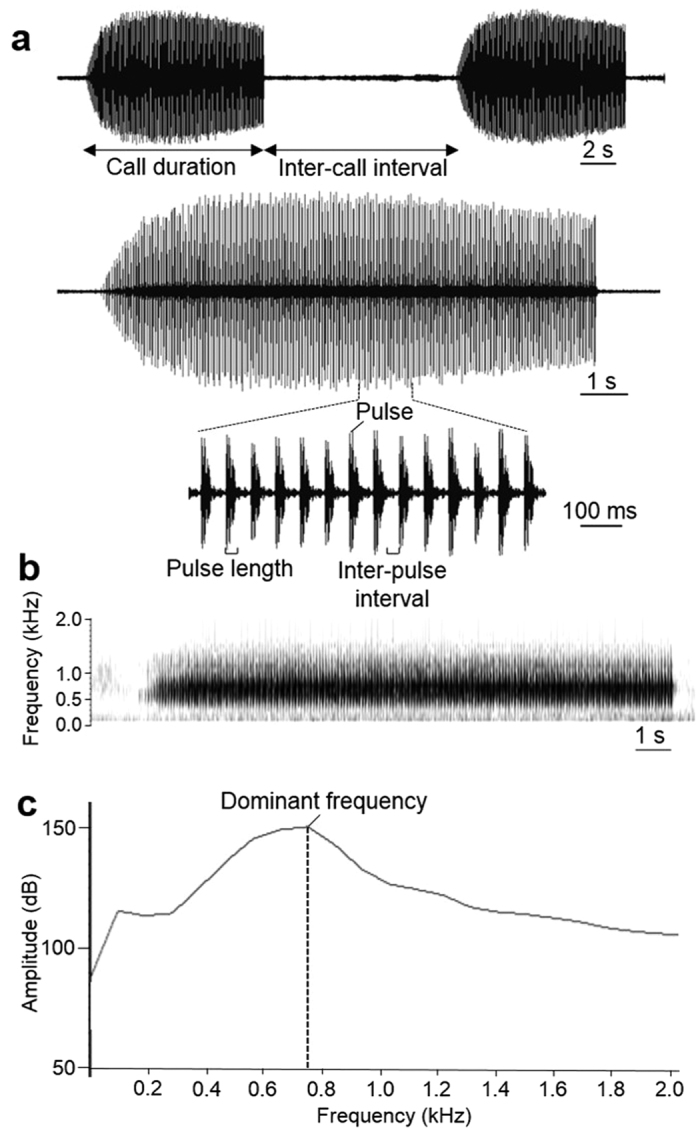
General description of advertisement calls of *R. marina* recorded in Townsville, Queensland, Australia at an air temperature of 20.0 °C. All figures were created in Raven Pro 1.4. (**a**) Oscillograms of two consecutive calls and a single call, and a close-up showing pulses in a call. The dimensions correspond to amplitude (milliunits, vertical) and time (sec, horizontal). This figure includes call duration (sec) and inter-call interval length (sec) in two calls and pulse numbers, pulse length (ms) and inter-pulse interval length (ms) in a single call. (**b**) Spectrogram of the same single call. The vertical dimension shows frequency in kilohertz (kHz) and the horizontal shows time (sec). Colour of the spectrogram indicates the relative intensity of the sound and frequency. (**c**) Power spectrum of that call (same setting as above) with amplitude (dB) and frequency (kHz) axes. Dominant frequency (kHz) is determined as the frequency at the highest amplitude.

Recordings were analysed using Raven Pro 1.4 (Cornell Lab of Ornithology, Ithaca, USA) and Avisoft SASLab Pro 5.2.02 (Avisoft Bioacoustics, Berlin, Germany) to measure or calculate seven different call properties from each of 286 recorded advertisement calls from NEQ (range 2 to 16 calls per male, mean = 8.9 ± 2.7 SD), 95 calls from SQ (3 to 10 calls/male, 7.9 ± 2.2 SD), 202 calls from CQ (3 to 10 calls/male, 7.2 ± 2.2 SD), and 181 calls from WA (4 to 10 calls/male, 6.5 ± 1.6 SD). First we downsampled the sampling rate of each call 96 kHz to 44.1 kHz using r8brain v1.9, in order to reduce frequency grid spacing from 93.8 Hz to 43.1 Hz in Raven Pro, and thus obtain more detailed values of dominant frequency. We measured the following temporal variables of each recorded call from the waveform and the spectrogram (Fig. 3a): (1) call duration (section); (2) inter-call interval (section); (3) pulse duration (ms); and (4) inter-pulse interval (ms). We calculated (5) proportion of time spent calling by dividing the sum of call duration by the sum of call duration plus all inter-call intervals. We also counted the number of pulses per call (Fig. 3b), and calculated (6) pulse rate (number of pulses per sec) by dividing pulse number by call duration. We measured (7) the dominant frequency (Hz) using Raven Pro’s spectrogram function (1024 points fast-Fourier transform [FFT], overlap 75%, Hamming’s sampling window with a frequency resolution of 56 Hz; Fig. 3c). We obtained average values (mean ± SD) of each of the seven call parameters for each individual male.

### Geographic and climatic data

Distance in km between each location were determined using Google maps, and climate data were taken from the Australian Bureau of Meteorology databases (www.bom.gov.au/), using long term climate averages from weather stations close to the field sites. Climate variables used were; mean annual temperature, mean maximal and minimal temperatures, mean temperature warmest and coldest quarters, temperature seasonality (standard deviation of monthly means), mean annual rainfall, and rainfall seasonality (coefficient of variation in monthly means).

### Statistical analysis

All statistical analyses were performed using the software package R (version 3.1.2., R core development team 2014). Since body size of calling males and temperature often affect advertisement call traits^1^, we performed multiple regressions using each call variable as the dependent variable and body size (SUL) and body temperature as independent variables, across all male toads from all populations. We found that dominant frequency and pulse length were significantly related to body size (N = 100, *r*^*2*^ = 0.226, *F*_2,98_ = 29.91, *P* < 0.001 for dominant frequency, N = 100, *r*^*2*^ = 0.165, *F*_2,98_ = 20.56, *P* < 0.001 for pulse length; Supplemental Fig. S1), and that pulse rate and inter-pulse interval were significantly related to temperature (N = 100, *r*^*2*^ = 0.221, *F*_2,98_ = 29.06, *P* < 0.001 for pulse rate, N = 100, *r*^*2*^ = 0.117, *F*_2,98_ = 14.12, *P* < 0.001 for inter-pulse interval; Supplemental Fig. S1). To remove those effects, and thus remove the possibly confounding effects of body size and thermal environment from the effects of population, analyses to examine population effects were carried out on the residuals of each of those four variables from the applicable regression. Before those analyses, all variables were standardized to z-scores. Those z-scores were used for the following analyses.

To investigate differences of call characters among populations we initially performed a multivariate analysis of variance (MANOVA) including all seven call properties and residuals, with hypothesis testing via Wilks’ Lambda. We used the first two canonical discriminant axes defined by that MANOVA to create a biplot illustrating the degree and manner in which our four populations differed. Following the significant MANOVA, we used univariate analyses of variance (ANOVAs) to test for significant differences among populations for each of the call parameters; pairwise Tukey’s HSD tests were used to determine which populations differed significantly for each call parameter.

We calculated coefficients of variation (CV = 100% × (SD/mean)) for each call parameter within individuals (CVi), within populations (CVp), and across all populations (CVa) using the individual, population or grand mean and standard deviations^33^. Based on these CV values, we determined the ratio of overall (or among) populations and within-population variation as CVa/CVp.

To examine possible effects of geographic and climate differences on how toad calls differed among populations, we calculated Euclidean distance matrices among populations for geographic distance and for each of the eight climatic variables using the first two axes of principle component analysis (PCA) (the “vegdist” function in the “vegan” R package). We summarised the overall variation in toad call parameters using a similar technique by determining the Euclidean distances between the centroids (the first two axes, accounting for 93.1% of the variability) from the canonical discriminant space. This created three distance matrices (toad calls, climate, geographic distance), including all six between site comparisons. We then performed linear regressions to test for linear relationships between pairwise site distances in call variable space and pairwise distances in the geographic and climate variable spaces.

## Additional Information

**How to cite this article**: Yasumiba, K. *et al.* Rapid differentiation of sexual signals in invasive toads: call variation among populations. *Sci. Rep.* **6**, 28158; doi: 10.1038/srep28158 (2016).

## Supplementary Material

Supplementary Information

## Figures and Tables

**Table 1:** Descriptive statistics for advertisement calls and body sizes of cane toad (residuals mean ± SD) and for body condition (mean ± SD), and results of univariate analyses of variation in each call property and body condition among locations.

Variables	NEQ (N = 32)	SQ (N = 12)	CQ (N = 28)	WA (N = 28)	*P*-value
Call properties					
Dominant frequency	24.26 ± 51.98	−15.59 ± 36.91	−21.23 ± 39.71	0.19 ± 42.60	0.001
Call duration	8.89 ± 4.19	8.97 ± 2.38	8.58 ± 2.57	12.19 ± 4.79	0.002
Inter-call interval	13.42 ± 9.42	33.73 ± 45.48	16.91 ± 13.20	23.85 ± 17.91	0.017
Proportion of time spent calling	0.46 ± 0.10	0.35 ± 0.13	0.42 ± 0.11	0.44 ± 0.14	0.051
Pulse rate	0.33 ± 1.35	−0.39 ± 1.16	−0.16 ± 1.33	−0.05 ± 1.74	0.404
Pulse length	0.02 ± 0.20	−0.02 ± 0.06	0.10 ± 0.35	−0.11 ± 0.15	0.009
Inter-pulse interval	0.43 ± 1.45	−0.13 ± 0.57	0.10 ± 1.29	−0.54 ± 0.75	0.016
Body condition					
Body size	95.09 ± 7.85	89.20 ± 5.43	107.23 ± 7.10	103.25 ± 4.55	<0.001
Body temperature	27.53 ± 1.84	25.08 ± 1.83	27.74 ± 1.30	29.67 ± 1.35	<0.001

N = number of individuals.

**Table 2:** Coefficient of variation (%) in within-individual (CVi), within-population (CVp) and overall mean values (CVa) for seven call parameters from 100 males recorded from four localities.

Call property	CVi	CVp	CVa	CVa/CVp
Dominant frequency	2.83 ± 0.88	6.40 ± 1.30	8.25	1.29
Call duration	22.40 ± 3.50	35.70 ± 9.32	41.78	1.17
Inter-call interval	45.70 ± 27.52	89.53 ± 30.39	105.72	1.18
Proportion of time spent calling	24.37 ± 14.76	29.40 ± 7.18	28.29	0.96
Pulse rate	5.26 ± 0.74	10.01 ± 1.59	11.02	1.10
Pulse length	20.91 ± 7.96	26.82 ± 12.56	38.41	1.43
Inter-pulse interval	18.52 ± 6.13	15.56 ± 5.62	19.98	1.28

## References

[b1] Gerhardt H. C. & Huber F. Acoustic communication in insects and anurans: common problems and diverse solutions (University of Chicago Press, 2002).

[b2] Wells K. D. The ecology & behavior of amphibians. (The University of Chicago Press, 2007).

[b3] Martin W. F. Evolution of vocalisation in the genus Bufo. (Texas University Press, 1972).

[b4] Pfennig K. S., Rapa K. & McNatt R. Evolution of male mating behavior: male spadefoot toads preferentially associate with conspecific males. Behav. Ecol. Sociobiol. 48, 69–74 (2000).

[b5] Felton A., Alford R. A., Felton A. M. & Schwarzkopf L. Multiple mate choice criteria and the importance of age for male mating success in the microhylid frog. Cophixalus ornatus. Behav. Ecol. Sociobiol. 59, 786–795 (2006).

[b6] Cheong S., Yoo J.-H., Park S.-R. & Sung H.-C. Age estimation by skeletochronology and advertisement call variation in the black-spotted pond frog (*Rana nigromaculata*). Anim. Cells Syst. 17 (2013).

[b7] Nevo E. Adaptive variation in size of cricket frogs. Ecology 54, 1271–1281 (1973).

[b8] Wells K. D. & Schwartz J. J. Vocal communication in a neotropical treefrog, *Hyla ebraccata*: Advertisement calls. Anim. Behav. 32, 405–420 (1984).

[b9] Ryan M. J. & Wilczynski W. Evolution of intraspecific variation in the advertisement call of a cricket frog (*Acris crepitans*, Hylidae). Biol. J. Linn. Soc. 44, 249–271 (1991).

[b10] Castellano S., Rosso A., Doglio S. & Giacoma C. Body size and calling variation in the green toad (*Bufo viridis*). J. Zool. 248, 83–90 (1999).

[b11] Pröhl H., Hagemann S., Karsch J. & Höbel G. Geographic variation in male sexual signals in strawberry poison frogs (*Dendrobates pumilio*). Ethology 113, 825–837 (2007).

[b12] Klymus K. E., Humfeld S. C. & Gerhardt H. C. Geographical variation in male advertisement calls and female preference of the wide-ranging canyon treefrog, Hyla arenicolor. Biol. J. Linn. Soc. 107, 219–232 (2012).

[b13] Castellano S., Giacoma C. & Dujsebayeva T. Morphometric and advertisement call geographic variation in polyploid green toads. Biol. J. Linn. Soc. 70, 341–360 (2000).

[b14] Podos J. & Warren P. S. The evolution of geographic variation in birdsong. Adv. Stud. Behav. 37, 403–458 (2007).

[b15] Baraquet M., Grenat P., Salas N. & Martino A. Geographic variation in the advertisement call of *Hypsiboas cordobae* (Anura, Hylidae). Acta. Ethologica. 1–8 (2014).

[b16] Zug G. R. & Zug P. B. The marine toad, Bufo marinus: a natural history resume of native populations. (Smithsonian Institution Press Washington, DC, 1979).

[b17] Alford R., Cohen M., Crossland M., Hearnden M. & Schwarzkopf L. Population biology of Bufo marinus in northern Australia. (Supervising Scientist, 1995).

[b18] Phillips B. L., Brown G. P., Webb J. K. & Shine R. Invasion and the evolution of speed in toads. Nature 439, 803–803 (2006). 16482148 10.1038/439803a

[b19] Alford R. A., Brown G. P., Schwarzkopf L., Phillips B. L. & Shine R. Comparisons through time and space suggest rapid evolution of dispersal behaviour in an invasive species. Wildlife Res. 36, 23–28 (2009).

[b20] Phillips B. L., Brown G. P. & Shine R. Evolutionarily accelerated invasions: the rate of dispersal evolves upwards during the range advance of cane toads. J. Evolution. Biol. 23 (2010). 10.1111/j.1420-9101.2010.02118.x 20939838

[b21] Sabath M. D., Boughton W. C. & Simon E. Expansion of the range of the introduced toad *Bufo marinus* in Australia from 1935 to 1974. Copeia 1981, 676–680 (1981).

[b22] Letnic M., Webb J. K. & Shine R. Invasive cane toads (*Bufo marinus*) cause mass mortality of freshwater crocodiles (*Crocodylus johnstoni*) in tropical Australia. Biol. Conserv. 141, 1773–1782 (2008).

[b23] Kearney M. *et al.* Modelling species distributions without using species distributions: the cane toad in Australia under current and future climates. Ecography 31, 423–434 (2008).

[b24] Goodgame D. *Kimberly Toad Busters*. Available at: http://www.canetoads.com.au/. (Accessed: 4th March 2016) (2005).

[b25] Slade R. W. & Moritz C. Phylogeography of *Bufo marinus* from its natural and introduced ranges. Proc. R. Soc. Lond. B. 265, 769–777 (1998). 10.1098/rspb.1998.0359 PMC1689048 9628036

[b26] Estoup A. *et al.* Combining genetic, historical and geographical data to reconstruct the dynamics of bioinvasions: application to the cane toad *Bufo marinus*. Mol. Ecol. Resour. 10, 886–901 (2010). 21565099 10.1111/j.1755-0998.2010.02882.x

[b27] Gerhardt H. C. Female mate choice in treefrogs: static and dynamic acoustic criteria. Anim. Behav. 42, 615–635 (1991).

[b28] AmphibiaWeb Team. AmphibiaWeb: Information on amphibian biology and conservation. Available at: http://amphibiaweb.org/. (Accessed: 4th March 2016) (2000).

[b29] O’Neill E. M. & Beard K. H. Clinal variation in calls of native and introduced populations of *Eleutherodactylus coqui*. Copeia 2011, 18–28 (2011).

[b30] Gleason J. M. & Ritchie M. G. Evolution of courtship song and reproductive isolation in the *Drosophila willistoni* species complex: do sexual signals diverge the most quickly? Evolution 52, 1493–1500 (1998). 28565374 10.1111/j.1558-5646.1998.tb02031.x

[b31] Llewelyn J., Phillips B., Alford R., Schwarzkopf L. & Shine R. Locomotor performance in an invasive species: cane toads from the invasion front have greater endurance, but not speed, compared to conspecifics from a long-colonised area. Oecologia 162, 343–348 (2010). 19841946 10.1007/s00442-009-1471-1

[b32] Brown G. P., Kelehear C. & Shine R. The early toad gets the worm: cane toads at an invasion front benefit from higher prey availability. J. Anim. Ecol. 82, 854–862 (2013). 23360501 10.1111/1365-2656.12048

[b33] Kaefer I. L. & Lima A. P. Sexual signals of the Amazonian frog *Allobates paleovarzensis*: geographic variation and stereotypy of acoustic traits. Behaviour 149, 15–33 (2012).

