# Supplementary material for: Rapid differentiation of sexual signals in invasive toads: call variation among populations
Source: Sci Rep. 2016 Jun 22;6:28158. doi: 10.1038/srep28158 (PMC4916444; doi:10.1038/srep28158)
Supplement: Supplementary Information [file srep28158-s1.pdf]

Supplementary materials:

Rapid differentiation of sexual signals in invasive toads: call variation among populations

Kiyomi Yasumiba,[1*] Richard L. Duffy,[1] Scott A. Parsons,[1] Ross A. Alford[1] and Lin Schwarzkopf[1]

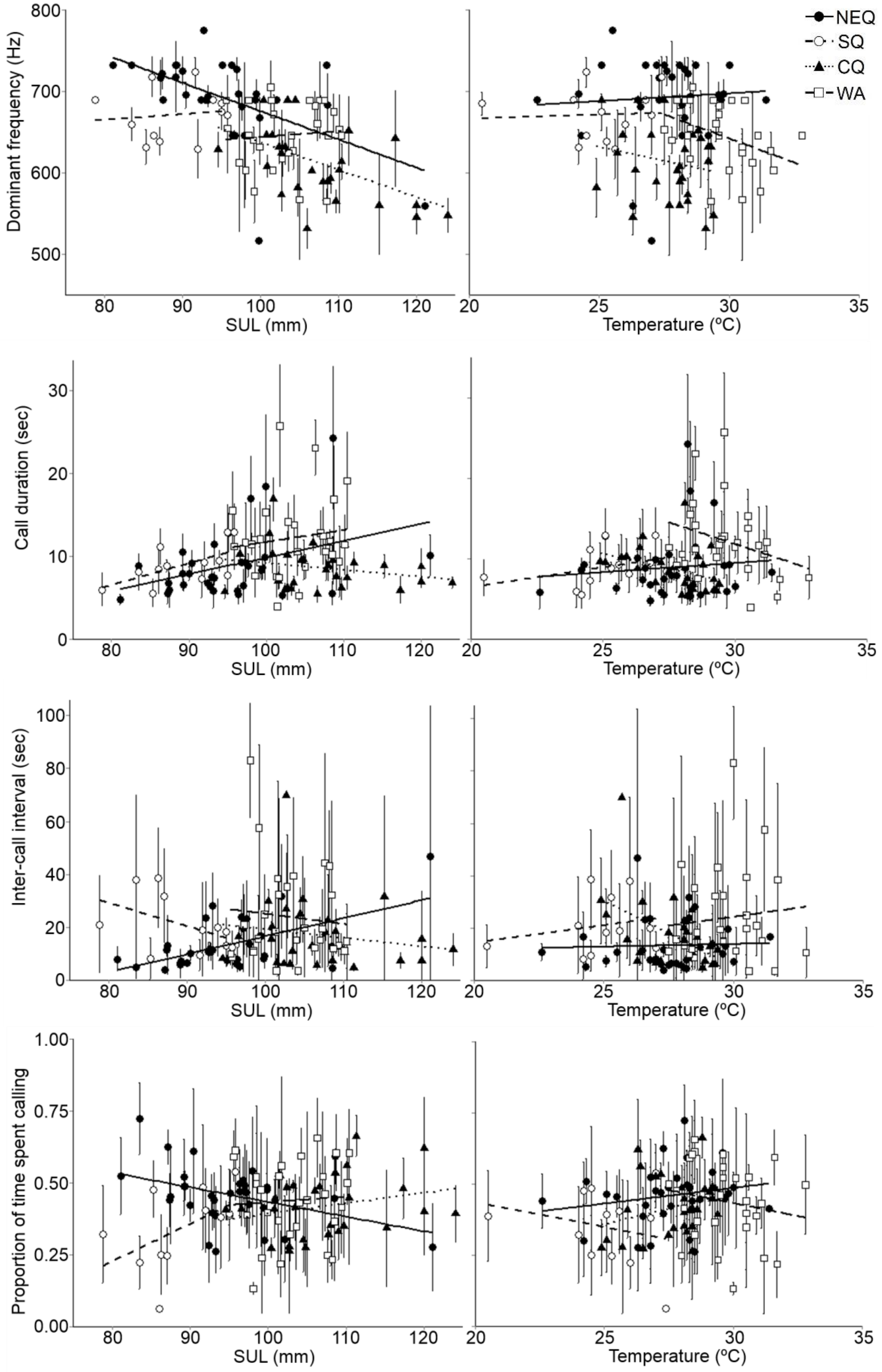
NEQ
SQ
CQ
WA
Dominant frequency (Hz)
Call duration (sec)
Inter-call interval (sec)
Proportion of time spent calling
SUL (mm)
Temperature (ºC)

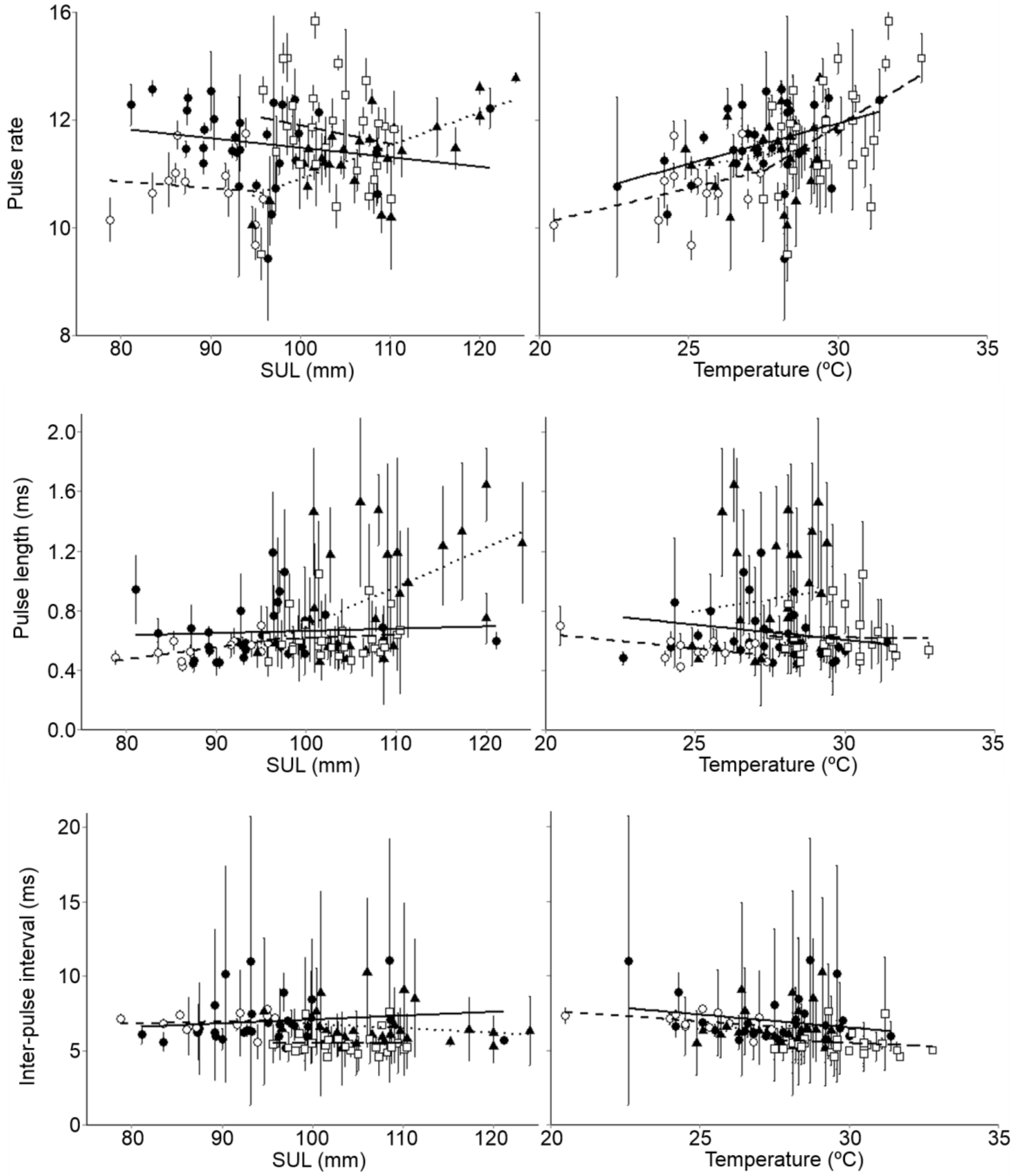

Supplementary Figure S1 Linear correlations of body size (SUL, mm) and temperature (°C) with each call parameter in four different populations; NEQ (solid line, solid circles), SQ (dashed line, open circles), CQ (dotted line, solid triangles) and WA (long dashed line, open squares).
